# OnabotulinumtoxinA is now an important tool for managing pediatric neurogenic lower urinary tract dysfunction

**DOI:** 10.3389/fped.2024.1407009

**Published:** 2024-06-03

**Authors:** Brendan T. Frainey, Douglass B. Clayton

**Affiliations:** Department of Urology, Division of Pediatric Urology, Monroe Carell Jr. Children’s Hospital at Vanderbilt, Vanderbilt University Medical Center, Nashville, TN, United States

**Keywords:** neurogenic bladder, myelomeningocele, pediatric urology, management, onabotulinumtoxinA

## Abstract

Initial urologic management of pediatric neurogenic lower urinary tract dysfunction (NLUTD) includes clean intermittent catheterization (CIC) regimen and use of anticholinergic or beta3 agonist medications. Historically, NLUTD that did not respond to these initial management strategies received open surgical procedures such as augmentation cystoplasty (AC) to increase bladder capacity and create a lower-pressure reservoir. Since its first reported use in 2002, intradetrusor onabotulinumtoxinA (BTX-A) injections has developed an emerging role in management of pediatric NLUTD, culminating in its recent FDA-approval in 2021. In this review, the current evidence regarding the safety, tolerability, and efficacy of BTX-A use in pediatric NLUTD will be summarized. Additionally, we will attempt to define the current role of BTX-A in the management of patients with NLUTD, discuss limitations to the current body of literature, and suggest future avenues of study.

## 1 Introduction

Neurogenic lower urinary tract dysfunction (NLUTD) describes dysfunction of the bladder and urethra due to a clinically confirmed neurological disorder (1, 2). In children, the majority of NLUTD is a result of myelomeningocele, a severe form of spina bifida (SB), but can be associated with other congenital (e.g., cerebral palsy) and acquired (e.g., spinal cord injury) neurologic conditions (3). Aside from surveillance with renal ultrasound, the initial urologic management of NLUTD includes clean intermittent catheterization (CIC) regimens and use of anticholinergic or beta3 agonist medications. These interventions are performed in conjunction with close monitoring of the upper and lower urinary tract with renal ultrasound, urodynamics, and serum creatinine (4, 5). However if conservative measures fail, additional treatments aimed at preserving upper urinary tract function and promoting continence may be warranted.

OnabotulinumtoxinA (BTX-A) is a neurotoxin, derived from the bacteria *Clostridium botulinum*, which is taken up by presynaptic efferent nerves where it cleaves the SNARE protein, SNAP-25, inhibiting the release of acetylcholine at the neuromuscular junction and resulting in reduced muscle spasticity (6–8). Since its first reported use in pediatric patients in 2002, intradetrusor BTX-A injection has become an increasingly common tool in bladder management for patients with neurogenic detrusor overactivity (NDO), culminating in its recent FDA approval in 2021 for pediatric NDO (9, 10).

Historically, refractory NLUTD was managed with open surgical procedures such as cutaneous vesicostomy, ileal conduit, and augmentation cystoplasty (AC). The two former procedures seek to lower bladder pressure through urinary diversion while AC increases bladder capacity and creates a lower-pressure reservoir, protecting the upper urinary tract. Despite its increasing popularity, the optimal role for the use of BTX in pediatric NLUTD remains undefined. In this review, we will summarize the current evidence regarding the safety, tolerability, and efficacy of BTX-A use in pediatric NLUTD. Additionally, we hope to provide clinicians with guidance on the current role of BTX-A in the management of patients with NLUTD in addition to future uses and alternative administrations strategies.

## 2 Methods

A comprehensive review was performed using PubMed and the Cochrane Library for all relevant articles from 2000 to March 2024, with an emphasis on more recent publications. The search terms “neurogenic bladder”, “onabotulinumtoxinA”, “pediatrics” and “myelomeningocele” were used for this review. Articles were excluded if they involved adult patients or lacked clear measures for safety or efficacy (clinical or urodynamic outcomes) within the article.

## 3 Safety, dosing and tolerability

The safety and tolerability of BTX-A was established in adults with NLUTD following two double blinded, placebo-controlled, multi-center randomized control trials (RCT) by Cruz et al. and Ginsberg et al. (11, 12). Both studies demonstrated that BTX-A injections were well-tolerated with adverse events (AEs) primarily limited to localized urologic events such as urinary tract infection (UTI), hematuria, and increased risk for CIC/urinary retention in those who voided spontaneously prior to BTX-A. Due to the favorable safety profile and efficacy, 200 U BTX-A was approved in 2011 for adults with urinary incontinence (UI) due to NDO who had failed anticholinergic therapy.

While studies reporting the off-label use of intradetrusor BTX-A in pediatric patients began as early as 2002, BTX-A did not obtain FDA approval for pediatric NDO until 2021. FDA approval occurred after a multicenter, double-blind RCT by Austin et al. The study included children aged 5–17 with NDO and urinary incontinence (UI) receiving either a single dose of 50, 100, or 200 U of BTX-A (maximum dose not to exceed 6 U/kg) (10). Over the 48-week follow-up period, serious adverse events were reported in 6.7%–10.5% of patients. UTI was the most common AE reported (29%) over the study period with other common AEs including headache (5.3%), gastroenteritis (4.4%), nasopharyngitis (3.5%), and diarrhea (3.5%). However, a sub-analysis of the annualized UTI rates in the 6 months leading up to the study compared to the actual study period were comparable (10).

In 2023, an extension study of the Austin et al. RCT (NCT01852058) was published assessing the long-term safety and tolerability of BTX-A over a median of 82 weeks (13). The safety profile of BTX-A was similar across all dosing groups and in the setting of repeated treatments. UTI was again the most reported AE (31%) with 3 serious treatment emergent AEs (3.2%) reported during the study, all of which were a result of UTI. No reported cases of autonomic dysreflexia, neutralizing antibody detection or distant spread of toxin occurred with the use of BTX-A for pediatric NDO. Because of the favorable long-term safety profile from these trials, the FDA approved BTX-A for pediatric NDO in children 5 years or older with dosing of 6 U/kg for children under 34 kg and 200 U for children over 34 kg. Notably, the 6 U/kg dosing approved by the FDA was significantly lower than prior non-randomized studies, which utilized doses ranging between 10 and 12 U/kg (14).

## 4 Efficacy

While early reports characterizing the efficacy of BTX-A in pediatric patients with NLUTD have been encouraging, the majority of studies are single institution series with no control group and variable primary endpoints and definitions of treatment success (9, 15–20). Therefore, conclusions regarding the utility of BTX-A must be made in this context. Most studies do assess urodynamic changes pre- and post-BTX while others examine clinical outcomes such as improvement in daytime incontinence episodes and/or avoidance of reconstructive procedures such as bladder augmentation. Additionally, the heterogeneity of patient cohorts and lack of long-term follow-up limit most studies. Table 1 summarizes some of the major studies regarding outcomes/efficacy.

**Table 1:** Key studies reporting efficacy of onabotulinumtoxinA.

Study	*N*	Population	Age (years)	Dosing	follow-up	Key findings/outcomes
Schulte-Baukloh et al. (9)	17	Spina bifida with NDO + Pdet_max_ >40 mmHg	10.8	12 U/kg (Max 300 U) Single injection	2–4 weeks	Capacity improved – 56% Pdet_max_ lowered – 33% Compliance increased – 122%
Figueroa et al. (21)	17	Spina bifida	10.7	10 U/kg (Max 300 U) Minimum 1 injection	48 months	Capacity improved – 27%; Compliance increased – 45% Continued improvement with multiple injections Improved symptoms – 77% (10/13) 87.5% avoided reconstruction
Marte (20)	47	Spina bifida	9.0	200 U (Maxx 12 U/kg) Minimum 1 injection	64 months	Leak point volume (LPV) improved – 66% No change in DLPP 81% dry between CIC
Tiryaki et al. (22)	16	Spina bifida	6.5	10 U/kg (Max 360 U) Single injection (13/16)	31 months	55% (5/9) of “primary NDO” subjects completely dry + UDS improvement 0% (0/7) of “fibrotic bladders” improved clinically or on UDS
Naqvi et al. (17)	30	“Neurogenic bladder”	7.4	40 U/kg (Dysport) Single injection (15/30)	Not listed	Capacity improved – 43%; NDO with 47% resolution rate No significant improvement in UDS parameters between 1st and last injection Significant improvement in compliance in the “low compliance cohort” (+10 cm/H_2_O) vs. “OAB cohort “ (+3.9 cm/H_2_O)
Austin et al. (10)	100	Spina bifida, Spinal cord injury, Transverse myelitis	11.3	50 U, 100 U, or 200 U (Max 6 U/kg) Single injection	48 weeks	Sig improvement in daytime UI episodes across all dose groups: −1.3/day Majority with improvement on patient reported scale: 75–81% Significant improvements in capacity, pDet_max_, and NDO rates
Bowen et al. (16)	39	“Neurogenic bladder”	10	10 U/kg (Max 200 U) Multiple injections	65 months	Downgrading of “risk grade” in 38% and 63% of patients using the NSBPR and Lurie Children's risk classifications on post-BTX UDS 16/26 (61.5%) proceeded to BA at a median of 36 months
Franco et al. (13)	95	Spina bifida, Spinal cord injury	12	50 U, 100 U, or 200 U (Max 6 U/kg) Multiple injections	82 weeks	Improvements in treatment UI episodes across all 3 treatment cycles for 200 U: −1.4 to −1.8 /day Improvements in capacity, Pdet_max_, and NDO across at 3 treatment cycles for 200 U

### 4.1 Urodynamic changes

Pre and post BTX-A urodynamics show BTX-A injection leads to reproducible changes in key urodynamic parameters including bladder capacity, maximum detrusor pressures (Pdet_max_), compliance and NDO prevalence. In a 2017 systematic review of 12 studies and 293 pediatric patients with spina bifida, Hascoet et al. reported significant reduction in Pdet_max_ (32%–54%), increase in cystometric capacity (27%–162%), and improvement in compliance (28%–180%) on post-BTX urodynamic assessment in a majority of published series (23). In the single prospective randomized trial assessing the safety and efficacy of 50 U, 100 U and 200 U of BTX-A, dose-dependent improvements occurred in functional bladder capacity and maximal detrusor pressure (Pdet_max_) from baseline at 6 weeks post-injection, with 200 U giving greater improvement relative to 50 U (10). Additionally, the percentage of patients demonstrating NDO decreased significantly compared to baseline (88%–94% to 45%–62%), with the greatest reductions reported in the 100 U and 200 U groups. In the extension study, these urodynamic improvements remained durable throughout 3 dose cycles (injection procedures) in patients receiving 200 U (13).

### 4.2 Clinical outcomes

When assessing clinical outcomes, improvement in continence and avoidance of reconstructive procedures are the two most cited endpoints. BTX-A reliably reduces daytime urinary incontinence (UI) with Austin et al. reporting 1.3 fewer incontinence episodes per day at 6 weeks post-BTX across multiple dosing regimens (10). Continued improvements were seen in the extension study with repeated injections of BTX-A at 200 U resulting in further reduction of daytime UI from baseline (−1.8 episodes/day) (13). Other non-randomized studies report “complete resolution” of incontinence with rates ranging from 32%–100% in patients with some combination of NDO +/− poorly compliant bladders (23).

However, the literature remains unclear as to whether BTX-A reliably reduces the need for major bladder reconstruction. Figueroa et al. reported that only 18% (3/17) of subjects undergoing repeated BTX-A injections ultimately required augmentation cystoplasty (AC) over a four-year period, while Bowen et al. demonstrated that 61% ultimately proceeded to AC at a median of 36 weeks following initial BTX-A injections (16, 21). These studies attempt to elucidate whether BTX-A provides a durable “rehabilitative” effect or simply is a means of delaying higher-risk interventions. Larger, prospective studies have not assessed need for reconstruction, likely because AC is not an easily definable “outcome,” but rather is a complex, multi-faceted decision which is provider and family dependent. In our opinion, it remains unclear for which patients repeat Botox injections are a viable option for long-term management vs. part of a stepwise approach aiming to delay AC. Nonetheless, it is clear that many providers are using BTX-A prior to consideration of AC.

### 4.3 Use in the poorly compliant/“end-stage” bladder

Several studies have focused on whether BTX-A is effective in poorly compliant, high-pressure bladders. One study by Tiryaki compared 9 patients with NDO to 7 patients with poorly compliant, “fibrotic” bladders and noted significant improvements in capacity and compliance (4.7–8.6 ml/cmH_2_O) in the NDO group compared to no change in either capacity or compliance (3.1–3.5 ml/cmH_2_O) in the poorly-compliant group (22). They concluded that BTX-A is “useless” in low-compliance bladders despite a small cohort of patients of which the majority (81%) only received a single injection of BTX-A.

Prior studies utilizing animal models have demonstrated that BTX-A can reverse detrusor hyperplasia and fibrosis in both the early and late-stage development of NLUTD (24, 25). More recently, clinical studies have also supported these findings. Softness et al. demonstrated that low-compliance bladders (<10 ml/cmH_2_O) had significant improvement (+3 ml/cmH_2_O) relative to high-compliance bladders (>10 ml/cmH_2_O) with 35% of low compliance bladders being recategorized as high-compliance following BTX-A injection (18). Similarly, Naqvi et al. showed poorly compliant bladders demonstrated significant improvement in compliance as compared to an OAB/NDO cohort (+10.2 ml/cmH_2_O vs. +3.9 ml/cmH_2_O, *p* = 0.016) (17). Notably in the low compliance group in this study, 71% of subjects received multiple injections, suggesting that conclusions/determinations regarding efficacy of BTX-A made after a single injection may be premature.

### 4.4 Use in detrusor sphincter dyssynergia (DSD)

Detrusor sphincter dyssynergia (DSD) is a urodynamic finding in individuals with a neurologic condition describing bladder outlet obstruction secondary to involuntary contraction of the urethral and/or periurethral striated muscles in the presence of a detrusor contraction (26). The gold standard for diagnosis is via needle electromyography, but the majority of centers do not have this ability and utilize patch electrodes and/or fluoroscopy on videourodynamics to make this diagnosis (27). The first urologic use of BTX-A was in 1988 for the treatment of DSD in adult males with spinal cord injury (SCI) by injecting BTX-A into the external urethral sphincter (28). Additional studies in adults with SCI have shown improvements in a variety of urodynamic and clinical parameters including reductions in maximum detrusor pressure (Pdet_max_), maximum urethral closing pressure (MUCP), post-void residual (PVR), and incontinence episodes in individuals who received BTX-A injections for NDO + DSD or DSD alone (28–30).

Within the pediatric literature, however, the use of BTX-A for the treatment of DSD is quite limited (31–34). Typical injection doses for this indication have ranged from 50 U to 300 units. Currently, only one study exists that includes subjects who received intrasphincteric BTX-A for a neurologic etiology (31). In this single institution study over 10 years, 5 children underwent BTX-A injection for DSD and 2/5 (40%) had complete resolution of symptoms (improved uroflow, PVR <20, no UTI). Median response time was 4 months and only 1 individual received both intradetrusor and intrasphincteric injection (31) (Greer). Additional studies within the pediatric literature have focused on intrasphincteric BTX-A for individuals with dysfunctional voiding (non-neurogenic). These studies are also limited by their small sample sizes and retrospective nature, but do demonstrate encouraging improvements in PVRs and other subjective data (32–34). Overall, the current evidence does not support the routine use of intrasphincteric BTX-A for DSD in patients with NLUTD, but data from the adult SCI population and pediatric dysfunctional voiding populations are encouraging and warrants further study in a pediatric neurogenic population.

## 5 Patient selection and role of BTX-A

### 5.1 General considerations

In 2020, the Spina Bifida Association (SBA) released its guidelines for the urologic management of individuals with spina bifida. The primary goals for management are threefold: (1) maintain normal renal function (“protect the upper urinary tract”), (2) achieve urinary continence as early as socially acceptable, and (3) maximize urologic independence (35). Important secondary outcomes include elimination of hostile bladder dynamics via medical management and reduction/elimination of operative reconstruction of the bladder. Notably, the use of BTX-A is not mentioned in these guidelines. However, because BTX-A is a low-risk, minimally invasive procedure that can improve demonstrated urodynamic and clinical parameters in pediatric patients with NLUTD, BTX-A injections is an excellent option for achieving many of these key primary and secondary urologic goals identified by the SBA working group.

Early management of pediatric NLUTD includes the initiation of CIC and anticholinergic or B3 agonist medications for the treatment of urinary incontinence in the setting of upper tract changes, recurrent symptomatic UTI, or bladder hostility noted on UDS (35). Historically when these initial management strategies fail, “refractory NLUTD” was managed with open surgical procedures such as AC to increase bladder capacity and create a lower-pressure reservoir, ultimately protecting the upper urinary tract. However, the morbidity of these procedures is well known with prolonged hospital stays and a ten-year reoperation rate of 44% (36). Because of this, BTX-A use is increasing among pediatric urologists, which may prevent or delay need for AC (37). Prior population level studies support this, demonstrating stable or declining rates of AC in the setting of increased BTX-A use pediatric patients in the United States (38–41).

### 5.2 Appropriate timing to introduce BTX-A

Given the variety of urodynamic and clinical benefits of BTX-A, it can be introduced and utilized in a variety of ways. BTX-A can be introduced “early” in cases of NDO and/or worsening compliance/detrusor pressures with associated urinary incontinence to decrease spasticity and improve capacity, reducing UI episodes in combination with appropriate CIC intervals (10, 17, 20, 21). Alternatively, BTX-A can be introduced “late” in individuals with poor compliance (<10 ml/cmH_2_O), high Pdet_max_ (>40 cmH_2_O), or upper tract changes on videourodynamics or renal ultrasound to reduce bladder hostility. Unfortunately in the latter group, preprocedural predictors for BTX-A success have not been identified or defined although some studies have shown some success (16–18).

Therefore, it would be our groups preference to introduce BTX-A earlier in the management of NLUTD when able to prevent and reverse early bladder remodeling. However, this is not always feasible given rapid clinical deterioration for some patients or family preferences regarding the need for general anesthesia. BTX-A is an option for patients with higher risk bladder function, and injection may improve compliance by shifting pressure curves into a safer zone where timely CIC can reduce the exposure of the upper tracts to high pressures (16).

### 5.3 Role of repeated injections and when to progress to open surgical reconstruction

In most studies to date, the effects of only a single injection of BTX-A have been reported to make determinations regarding the efficacy of BTX-A in pediatric patients. Despite its relatively long duration of action, the efficacy of BTX-A wanes after 3–6 months requiring repeated injection for sustained effects (7). In adult patients with NDO, repeated injections are commonplace and the need for repeat procedures are often dictated by the recurrence of symptoms such as urinary incontinence.

In the pediatric literature, several studies have reported on the effects of repeat injections. In the single randomized prospective study by Franco et al., repeat injections with 200 U led to consistent improvements in daytime UI and high patient satisfaction (75%) based on a modified treatment benefit scale (13). Additionally, improvements in UDS parameters such as capacity, Pdet_max_, and proportion of patients experiencing NDO were noted throughout all 3 treatment cycles during the study period. In the study by Bowen et al., 39 subjects underwent 165 injections with the median number of injections per subject of 4 (IQR 3–7) (16). Of the 26 subjects deemed “at-risk” for AC, 16 (61.5%) went on to AC at a median of 36 months after a median of 4.5 injections prior to AC. Another study by Naqvi reported that 23% of patients ultimately required AC. In this study, significant improvements were seen in “low compliance” bladders with a median compliance change from 6.3 ml/cmH_2_O at baseline to 14.1 ml/cmH_2_O after BTX-A and consistent UDS improvements were maintained with repeat injections (17). Therefore, repeated injections should be considered for most patients unless there is no response/worsening on UDS and/or clinical parameters at first post-BTX evaluation.

More recent literature has focused on surgical decision-making surrounding the choice between BTX-A and AC. Li et al. performed a retrospective review of 14 patients who had AC and 50 patients who had BTX-A over a 5-year period and identified 10 key clinical factors that influenced management decisions (42). Surprisingly, they found that “desire for independence/continence” and “reduced bladder capacity” were significantly associated with AC whereas factors typically associated with bladder hostility were associated with BTX-A. This study highlights the importance of shared decision-making with patients and families regarding management decisions for pediatric NLUTD.

## 6 Discussion

Since its first reported use in children in 2002, the use of BTX-A in pediatric patients with NLUTD has increased rapidly over the past two decades culminating in its FDA-approval for pediatric NDO in 2021 (9, 10). FDA approval was largely due to the multi-center, prospective, double-blind randomized control trial by Austin et al. which demonstrated both the safety and efficacy of BTX-A across different dosing regimens (50 U, 100 U, 200 U), with UTI being the major safety concern (10). An extension study from the initial trial confirmed the long-term safety of BTX-A in pediatric patients and demonstrated consistent improvements with repeated injections in regard to incontinence episodes and urodynamic parameters (13). Much of the remaining literature consists of smaller, retrospective studies with heterogeneous study design, primary endpoints, and follow-up. Despite this, BTX-A objectively improves both urodynamic and clinical parameters for most patients with NLUTD and is a low-risk procedure.

While the benefits of BTX-A are clear, the profile of the NLUTD patient that derives the greatest benefit from this therapy remains poorly defined. Should BTX-A be introduced proactively prior to the development of hostile UDS or concerning imaging findings or should it be reserved until conservative measures like CIC and oral medications fail? When should BTX-A be abandoned in favor of surgical reconstruction? While gaps in the current literature remain, we believe that the choice to pursue BTX-A and/or AC should utilize a shared decision-making approach between patients, families and providers given the complexity and multi-factorial nature of these treatment choices. We recommend that BTX-A treatment is introduced to patients and caregivers early in the management of pediatric NLUTD. As supported by several studies, we further believe that BTX-A can be offered in the patient with a poorly compliant, hostile bladder.

Compared to lower urinary tract reconstruction, BTX-A costs less and carries low morbidity which makes it a very attractive option in a patient group that can be extraordinarily complex. Additionally, use of BTX-A may allow providers to stop or reduce the dose/frequency of anticholinergic medications which have several significant side effects such as constipation, dry mouth, dry eyes, and cognitive effects. However, when compared to CIC and oral medications, BTX-A requires general anesthesia and repeat injections due to the finite efficacy of each individual injection (43). Overland et al. recently demonstrated that awake injection of BTX-A can be performed in pediatric patients with NGB (44). While this requires a paradigm shift in expectations from families and clinic workflow, awake injections would reduce anesthetic exposures and operative costs for a group of patients already at risk for frequent anesthetic exposures over their lifetime. The future of chemodenervation for pediatric NLUTD may focus on identifying alternative therapies to BTX-A such as novel engineered chimeric botulinum-like toxins, such as BoNT/B_my-ww_, which may have improved efficacy and lower side-effect profiles due to their unique binding properties (45). Another promising avenue of study has focused on development of alternative modalities for BTX-A administration, specifically liposome-encapsulated BTX-A, which may provide a lower-risk alternative to injection therapy and unique drug-delivery mechanism (46).

In conclusion, BTX-A has become a central therapeutic option in the management of pediatric NLUTD. While its urodynamic and clinical benefits have been demonstrated, future studies should be aimed at determining which patients benefit most and when therapy should be introduced.

## References

[1] Gajewski JB Drake MJ. Neurological lower urinary tract dysfunction essential terminology. Neurourol Urodyn. (2018) 37(S6):S25–31. 10.1002/nau.23758 30614062

[2] Drake MJ Apostolidis A Cocci A Emmanuel A Gajewski JB Harrison SCW Neurogenic lower urinary tract dysfunction: clinical management recommendations of the neurologic incontinence committee of the fifth international consultation on incontinence 2013. Neurourol Urodyn. (2016) 35(6):657–65. 10.1002/nau.23027 27176559

[3] Sager C Barroso U Netto JMB Retamal G Ormaechea E. Management of neurogenic bladder dysfunction in children update and recommendations on medical treatment. Int Braz J Urol. (2022) 48(1):31–51. 10.1590/s1677-5538.ibju.2020.0989 33861059 PMC8691255

[4] Routh JC Cheng EY Austin JC Baum MA Gargollo PC Grady RW Design and methodological considerations of the centers for disease control and prevention urologic and renal protocol for the newborn and young child with spina bifida. J Urol. (2016) 196(6):1728–34. 10.1016/j.juro.2016.07.081 27475969 PMC5201100

[5] Snow-Lisy DC Yerkes EB Cheng EY. Update on urological management of spina bifida from prenatal diagnosis to adulthood. J Urol. (2015) 194(2):288–96. 10.1016/j.juro.2015.03.107 25839383

[6] Hughes AJ. Botulinum toxin in clinical practice. Drugs. (1994) 48(6):888–93. 10.2165/00003495-199448060-00005 7533696

[7] Dong M Masuyer G Stenmark P. Botulinum and tetanus neurotoxins. Annu Rev Biochem. (2019) 88:811. 10.1146/annurev-biochem-013118-111654 30388027 PMC7539302

[8] Dolly JO Lawrence GW. Chapter 3: molecular basis for the therapeutic effectiveness of botulinum neurotoxin type A. Neurourol Urodyn. (2014) 33(Suppl 3):S14–20. 10.1002/nau.22634 25042137

[9] Schulte-Baukloh H Michael T Schobert J Stolze T Knispel HH. Efficacy of botulinum—a toxin in children with detrusor hyperreflexia due to myelomeningocele: preliminary results. Urology. (2002) 59(3):325–7. 10.1016/S0090-4295(01)01641-7 11880062

[10] Austin PF Franco I Dobremez E Kroll P Titanji W Geib T Onabotulinumtoxina for the treatment of neurogenic detrusor overactivity in children. Neurourol Urodyn. (2021) 40(1):493. 10.1002/nau.24588 33305474 PMC7839517

[11] Cruz F Herschorn S Aliotta P Brin M Thompson C Lam W Efficacy and safety of onabotulinumtoxinA in patients with urinary incontinence due to neurogenic detrusor overactivity: a randomised, double-blind, placebo-controlled trial. Eur Urol. (2011) 60(4):742–50. 10.1016/j.eururo.2011.07.002 21798658

[12] Ginsberg D Gousse A Keppenne V Sievert KD Thompson C Lam W Phase 3 efficacy and tolerability study of onabotulinumtoxinA for urinary incontinence from neurogenic detrusor overactivity. J Urol. (2012) 187(6):2131–9. 10.1016/j.juro.2012.01.125 22503020

[13] Franco I Hoebeke PB Dobremez E Titanji W Geib T Jenkins B Long-term safety and tolerability of repeated treatments with onabotulinumtoxinA in children with neurogenic detrusor overactivity. J Urol. (2023) 209(4):774–84. 10.1097/JU.0000000000003157 36655470 PMC12721605

[14] Gamé X Mouracade P Chartier-Kastler E Viehweger E Moog R Amarenco G Botulinum toxin-A (Botox®) intradetrusor injections in children with neurogenic detrusor overactivity/neurogenic overactive bladder: a systematic literature review. J Pediatr Urol. (2009) 5(3):156–64. 10.1016/j.jpurol.2009.01.005 19264554

[15] Khan MK VanderBrink BA DeFoor WR Minevich E Jackson E Noh P Botulinum toxin injection in the pediatric population with medically refractory neuropathic bladder. J Pediatr Urol. (2016) 12(2):104.e1–e6. 10.1016/j.jpurol.2015.08.018 26778185

[16] Bowen DK Meyer T Rosoklija I Sturm R Chu DI Cheng EY Botulinum toxin in patients at-risk for bladder augmentation: durable impact or kicking the can? Neurourol Urodyn. (2022) 41(6):1406–13. 10.1002/nau.24962 35670258

[17] Naqvi S Clothier J Wright A Garriboli M. Urodynamic outcomes in children after single and multiple injections for overactive and low compliance neurogenic bladder treated with abobotulinum toxin A. J Urol. (2020) 203(2):413–9. 10.1097/JU.0000000000000540 31518199

[18] Softness KA Thaker H Theva D Rajender A Cilento BG Bauer SB. Onabotulinumtoxin A (botox): a reasonable alternative for refractory neurogenic bladder dysfunction in children and young adults. Neurourol Urodyn. (2021) 40(8):1981–8. 10.1002/nau.24778 34486166 PMC8556295

[19] Le Nué R Harper L De Sèze M Bouteiller C Goossens D Dobremez E. Evolution of the management of acquired neurogenic bladder in children using intradetrusor botulinum toxin type A injections: 5-year experience and perspectives. J Pediatr Urol. (2012) 8(5):497–503. 10.1016/j.jpurol.2011.09.014 22115699

[20] Marte A. Onabotulinumtoxin A for treating overactive/poor compliant bladders in children and adolescents with neurogenic bladder secondary to myelomeningocele. Toxins (Basel). (2012) 5(1):16–24. 10.3390/toxins5010016 23274271 PMC3564065

[21] Figueroa V Romao R Pippi Salle JL Koyle MA Braga LHP Bägli DJ Single-center experience with botulinum toxin endoscopic detrusor injection for the treatment of congenital neuropathic bladder in children: effect of dose adjustment, multiple injections, and avoidance of reconstructive procedures. J Pediatr Urol. (2014) 10(2):368–73. 10.1016/j.jpurol.2013.10.011 24280272

[22] Tiryaki S Yagmur I Parlar Y Ozel K Akyildiz C Avanoglu A Botulinum injection is useless on fibrotic neuropathic bladders. J Pediatr Urol. (2015) 11(1):27.e1–e4. 10.1016/j.jpurol.2014.08.009 25448589

[23] Hascoet J Manunta A Brochard C Arnaud A Damphousse M Menard H Outcomes of intra-detrusor injections of botulinum toxin in patients with spina bifida: a systematic review. Neurourol Urodyn. (2017) 36(3):557–64. 10.1002/nau.23025 27187872

[24] Tinay I Tanidir Y Cikler E Cetinel S Tarcan T. Intradetrusor botulinum neurotoxin A (BoNT-A) injections decrease bladder fibrosis secondary to partial urethral obstruction in the male rat model. Neurourol Urodyn. (2012) 31(4):564–70. 10.1002/nau.21248 22275224

[25] Temeltas G Tikiz C Dagci T Tuglu I Yavasoglu A. The effects of botulinum-A toxin on bladder function and histology in spinal cord injured rats: is there any difference between early and late application? J Urol. (2005) 174(6):2393–6. 10.1097/01.ju.0000180410.78774.b5 16280854

[26] Bauer SB Nijman RJM Drzewiecki BA Sillen U Hoebeke P. International children’s continence society standardization report on urodynamic studies of the lower urinary tract in children. Neurourol Urodyn. (2015) 34(7):640–7. 10.1002/nau.22783 25998310

[27] Stoffel JT. Detrusor sphincter dyssynergia: a review of physiology, diagnosis, and treatment strategies. Transl Androl Urol. (2016) 5(1):127. Available online at: https://www.ncbi.nlm.nih.gov/pmc/articles/PMC4739973/ (cited April 30, 2024). 26904418 10.3978/j.issn.2223-4683.2016.01.08 PMC4739973

[28] Dykstra DD Sidi AA Scott AB Pagel JM Goldish GD. Effects of botulinum A toxin on detrusor-sphincter dyssynergia in spinal cord injury patients. J Urol. (1988) 139(5):919–22. 10.1016/S0022-5347(17)42717-0 3361663

[29] Chen SL Bih LI Huang YH Tsai SJ Lin TB Kao YL. Effect of single botulinum toxin A injection to the external urethral sphincter for treating detrusor external sphincter dyssynergia in spinal cord injury. J Rehabil Med. (2008) 40(9):744–8. 10.2340/16501977-0255 18843427

[30] Schulte-Baukloh H Weiß C Stolze T Herholz J Stürzebecher B Miller K Botulinum-A toxin detrusor and sphincter injection in treatment of overactive bladder syndrome: objective outcome and patient satisfaction. Eur Urol. (2005) 48(6):984–90. 10.1016/j.eururo.2005.06.021 16126328

[31] Greer T Abbott J Breytenbach W McGuane D Barker A Khosa J Ten years of experience with intravesical and intrasphincteric onabotulinumtoxinA in children. J Pediatr Urol. (2016) 12(2):94.e1–6. 10.1016/j.jpurol.2015.06.019 26472538

[32] Vricella GJ Campigotto M Coplen DE Traxel EJ Austin PF. Long-Term efficacy and durability of botulinum-A toxin for refractory dysfunctional voiding in children. J Urol. (2014) 191(5):1586–91. 10.1016/j.juro.2013.10.034 24679879

[33] Radojicic ZI Perovic S V Milic NM. Is it reasonable to treat refractory voiding dysfunction in children with botulinum-A toxin? J Urol. (2006) 176(1):332–6. 10.1016/S0022-5347(06)00298-9 16753436

[34] Franco I Landau-Dyer L Isom-Batz G Collett T Reda EF. The use of botulinum toxin A injection for the management of external sphincter dyssynergia in neurologically normal children. J Urol. (2007) 178:1775–80. 10.1016/j.juro.2007.03.185 17707430

[35] Joseph DB Baum MA Tanaka ST Frimberger DC Misseri R Khavari R Urologic guidelines for the care and management of people with spina bifida. J Pediatr Rehabil Med. (2020) 13(4):479–89. 10.3233/PRM-200712 33252091 PMC7838970

[36] Szymanski KM Misseri R Whittam B Hollowell N Hardacker RE Swenson CR Additional surgeries after bladder augmentation in patients with spina bifida in the 21st century. J Urol. (2020) 203(6):1207–13. 10.1097/JU.0000000000000751 31951496

[37] Vasdev R Softness K Cahill D Panagides J Logvinenko T Saunders R Intradetrusor botox injection and augmentation cystoplasty trends among spina bifida patients at US freestanding children’s hospitals. J Pediatr Urol. (2024):S1477-5131(24)00092-5. 10.1016/j.jpurol.2024.02.012. [Epub ahead of print]. 38402080

[38] Schlomer BJ Saperston K Baskin L. National trends in augmentation cystoplasty in the 2000s and factors associated with patient outcomes. J Urol. (2013) 190(4):1352–8. 10.1016/j.juro.2013.04.075 23643599

[39] Lendvay TS Cowan CA Mitchell MM Joyner BD Grady RW. Augmentation cystoplasty rates at children’s hospitals in the United States: a pediatric health information system database study. J Urol. (2006) 176(4 Pt 2):1716–20. 10.1016/S0022-5347(06)00615-X 16945630

[40] Matta R Horns JJ Jacobson DL Schaeffer AJ Wallis MC Lau GA. National trends and outcomes in the use of intravesical botulinum toxin and enterocystoplasty among patients with myelomeningocele. Urology. (2022) 166:289–96. 10.1016/j.urology.2022.04.020 35523288 PMC9844129

[41] Biers SM Venn SN Greenwell TJ. The past, present and future of augmentation cystoplasty. BJU Int. (2012) 109(9):1280–93. 10.1111/j.1464-410X.2011.10650.x 22117733

[42] Li B Peard LM Zhao S Graham MK Adams C Taylor AS Understanding factors influencing primary treatment with intradetrusor onabotulinumtoxinA versus augmentation cystoplasty in patients with spina bifida. Neurourol Urodyn. (2023) 42(6):1431–6. 10.1002/nau.25219 37249147

[43] Dyer LL Franco I. Botulinum toxin-A therapy in pediatric urology: indications for the neurogenic and non-neurogenic neurogenic bladder. Sci World J. (2009) 9:1300–5. 10.1100/tsw.2009.146 PMC5823131 19936566

[44] Overland MR Leva NV Disandro M Copp HL. Feasibility of awake intravesical botulinum toxin injection in pediatric neurogenic bladder. J Urol. (2022) 208(3):702–10. 10.1097/JU.0000000000002709 35446131

[45] Thaker H Zhang S Diamond DA Dong M. Beyond botulinum neurotoxin A for chemodenervation of the bladder. Curr Opin Urol. (2021) 31(2):140. 10.1097/MOU.0000000000000843 33394765 PMC7983298

[46] Hung FC Kuo HC. Liposome-encapsulated botulinum toxin A in treatment of functional bladder disorders. Toxins (Basel). (2022) 14(12):838. 10.3390/toxins14120838 36548734 PMC9781836

